# Protective Effect of Ischemic Postconditioning against Ischemia Reperfusion-Induced Myocardium Oxidative Injury in IR Rats

**DOI:** 10.3390/molecules17043805

**Published:** 2012-03-27

**Authors:** Li Zhang, Jiangwei Ma, Huajin Liu

**Affiliations:** Department of Cardiology, Fengxian Branch of Shanghai 6th People’s Hospital, Shanghai 201400, China

**Keywords:** ischemia-reperfusion, ischemic postconditioning, myocardium, oxidative injury

## Abstract

Brief episodes of myocardial ischemia-reperfusion (IR) employed during reperfusion after a prolonged ischemic insult may attenuate the total ischemia-reperfusion injury. This phenomenon has been termed ischemic postconditioning. In the present study, we studied the possible effect of ischemic postconditioning on an ischemic reperfusion (IR)-induced myocardium oxidative injury in rat model. Results showed that ischemic postconditioning could improve arrhythmia cordis, reduce myocardium infarction and serum creatin kinase (CK), lactate dehydrogenase (LDH) and aspartate transaminase (AST) activities in IR rats. In addition, ischemic postconditioning could still decrease myocardium malondialdehyde (MDA) level, and increased myocardium Na^+^-K^+^-ATPase, Ca^2+^-Mg^2+^-ATPase, superoxide dismutase (SOD), catalase (CAT), glutathione peroxidase (GSH-Px) and glutathione reductase (GR) activities. It can be concluded that ischemic postconditioning possesses strong protective effects against ischemia reperfusion-induced myocardium oxidative injury in IR rats.

## 1. Introduction

Despite the powerful protective effects of ischemic preconditioning, the clinical application of this phenomenon has been rather disappointing, mainly because preconditioning must be instituted before the ischemic event [1,2,3]. In contract, there is a more promising approach to cardioprotection termed “ischemic postconditioning” (IPO). Ischemic postconditioning (IPO) was described for the first time in dogs by Zhao *et al*. [4]. These authors demonstrated that a series of repetitive cycles of brief reperfusion and reocclusion of the coronary artery applied at the onset of reperfusion after a prolonged ischemic event reduced infarct size. This finding has been confirmed in numerous investigations performed in mice [5,6], rats [7,8,9], rabbits [10,11,12,13], pigs [14,15,16], and humans [17,18,19]. To date, different mechanisms have been associated with this cardioprotective strategy [20,21,22], including the involvement of various G protein-coupled membrane receptors [23,24] and protein kinases [25].

In spite of the advances in myocardial protection with cardioplegia, myocardial tissue subjected to a period of ischemia undergoes morphological and functional damage, which increases during the reperfusion phase [23,24,25,26,27,28]. Reperfusion of ischaemic heart increases the detrimental effect of early ischemic injury by the release of reactive oxygen species (ROS). ROS sources include the electron transport chain, oxidant enzymes such as xanthine oxidase, and phagocytes. ROS may cause cellular damage by peroxidation of membrane lipids, sulfhydryl enzyme inactivation, protein cross-linking and DNA breakdown [26,29,30,31,32]. To counter this potential damage, organisms have enzymatic (such as superoxide dismutase (SOD), glutathione peroxidase (GSH-Px), catalase (CAT)) and nonenzymic (tocopherols, carotenes, ubiquinol, glutathione, ascorbic acid) antioxidant defences [27,33,34,35]. Oxidative stress occurs when there is either an over production of ROS or a decrease of antioxidant status. In this study, we investigate the protective effect of ischemic postconditioning on myocardium oxidative injury in IR rats.

## 2. Result

Table 1 shows the change of the ventricular arrhythmia of control and experimental animals. The value of ventricular tachycardia threshold (VTt) and ventricular arrhythmias scores (VAS) were significantly higher in IR rats than sham operation rats. In IPO group, the value of VTt and VAS were significantly lower as compared to IR rats.

**Table 1:** Effect of ischemic postconditioning on the value of VTt, VFt and VAS in IR rats.

Indexs	SO	IR	IPO
VTt (s)	2.63 ± 4.61	64.53 ± 21.86 **	22.18 ± 25.15 ^##^
VFt (s)	0	12.51 ± 20.15	0
VAS	0.62 ± 0.8	4.01 ± 1.19 **	2.43 ± 1.01 ^##^

** *p* < 0.01, IR group *vs*. SO group; ^##^ *p* < 0.01, IPO group *vs*. IR group. Entricular fibrillation threshold (VFt).

As shown in Table 2, there were no significant differences in initial values (R0min) of left ventricular systolic pressure (LVSP), left ventricular end diastolic pressure (LVEDP), and ±dp/dtmax between groups. The value of LVSP was significantly lower in IR and IPO rats than sham operation rats. In addition, the value of LVSP was significantly higher in IPO rats than IR rats. Moreover, the value of LVSP increased with extended reperfusion time in IPO rats. The value of LVEDP was significantly higher in IR and IPO rats than sham operation rats. In addition, the value of LVEDP was significantly lower in IPO rats than IR rats. The value of ±dp/dtmax was significantly lower in IR and IPO rats than sham operation rats. In addition, the value of ±dp/dtmax was significantly higher in IPO rats than IR rats.

**Table 2:** Effect of ischemic postconditioning on the value of LVSP, LVEDP, and ±dp/dtmax in IR rats.

Index		SO	IR	IPO
LVSP (mmHg)	Base	138.68 ± 14.29	141.05 ± 13.09	139.37 ± 12.17
	R15min	136.35 ± 15.29	80.24 ± 5.92	95.11 ± 7.93
	R30min	135.79 ± 11.69	86.03 ± 4.82 **	110.44 ± 12.84 ^##^
	R60min	127.82 ± 13.27	87.31 ± 9.03 **	112.73 ± 13.02 ^##^
	R120min	125.72 ± 11.05	69.08 ± 5.39 **	101.74 ± 12.18 ^##^
LVEDP (mmHg)	Base	−4.06 ± 2.31	5.13 ± 4.22 *	6.22 ± 3.17 ^#^
	R15min	−3.21 ± 2.16	16.02 ± 8.51 **	6.03 ± 4.27 ^##^
	R30min	−2.59 ± 1.62	18.39 ± 7.39 **	7.35 ± 3.02 ^##^
	R60min	−1.94 ± 1.58	20.11 ± 8.92 **	7.42 ± 2.99 ^##^
	R120min	−1.73 ± 2.01	23.15 ± 11.73 **	9.73 ± 3.17 ^##^
+dp/dtmax (mmHg/s)	Base	6,281.9 ± 2,900.5	6,301.7 ± 3,011.3	6,316.4 ± 2,794.1
	R15min	5,927.1 ± 2,176.4	3,517.2 ± 1,538.9 *	4,016.3 ± 1,933.5 ^#^
	R30min	5,827.8 ± 1,276.3	2,718.4 ± 893.2 *	4,083.2 ± 1,739.2 ^#^
	R60min	5,628.5 ± 2,007.4	2,561.7 ± 599.2 *	4,217.3 ± 2,175.4 ^#^
	R120min	5,513.1 ± 2,166.3	2,381.4 ± 739.1 *	3,977.2 ± 1,308.4 ^#^
−dp/dtmax (mmHg/s)	Base	5,132.8 ± 1,694.3	5,124.6 ± 1,352.7	5,341.7 ± 1,595.4
	R15min	5,044.4 ± 1,488.6	1,984.2 ± 674.4 **	3,106.3 ± 900.5 ^##^
	R30min	5,051.2 ± 1,276.4	2,264.2 ± 832.1 **	3,582.9 ± 1,254.3 ^##^
	R60min	4,885.9 ± 1,366.8	2,607.5 ± 927.7 **	3,606.3 ± 846.3 ^##^
	R120min	4,633.5 ± 1,298.1	2,287.9 ± 715.6 **	3,364.7 ± 947.9 ^##^

* *p* < 0.05, ** *p* < 0.01, IR group *vs*. SO group; ^#^ *p* < 0.05, ^##^ *p* < 0.01, IPO group *vs*. IR group.

**Figure 1:**
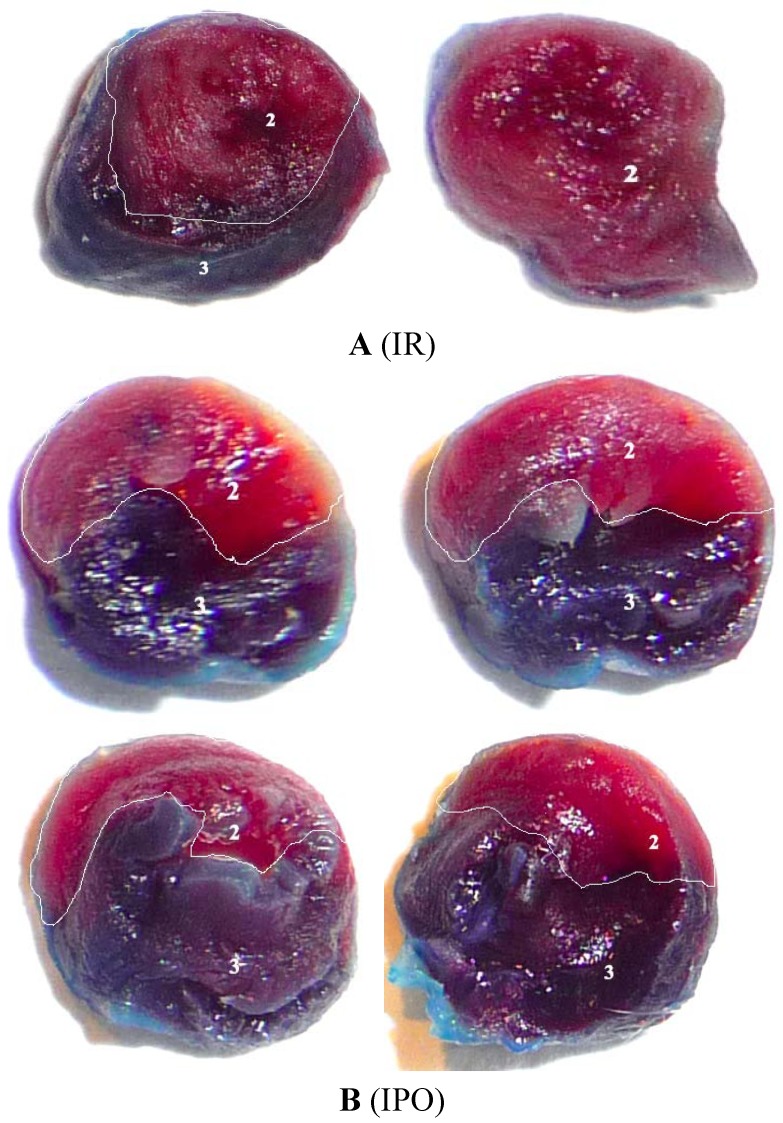
Effects of ischemic postconditioning on infarction.

Left ventricular (LV), area at risk (AAR), AAR/LV, myocardial infarction area (MIA) and MIA/AAR in rats data for all experimental groups are shown in the Table 3 and Figure 1. There were no significant differences in LV, AAR, AAR/LV between IR and IPO groups. The MIA and MIA/AAR of IPO rats were considerably lower than that of IR rats.

**Table 3:** Effect of ischemic postconditioning on the myocardium infarction in IR rats.

Indexes	SO	IR	IPO
LV	-	525.66 ± 47.13	516.65 ± 44.57
AAR	-	259.46 ± 24.16	207.24 ± 29.11
AAR/LV (%)	-	49.64 ± 5.87	40.47 ± 4.96
MIA	-	68.24 ± 5.73	36.31 ± 3.78 ^##^
MIA/AAR (%)	-	27.64 ± 1.98	17.68 ± 1.73 ^##^

^##^ *p* < 0.01, IPO group *vs*. IR group. The area of myocardial infarction (MIA).

Table 4 showed significant increased serum CK, LDH and AST activities in group IR as compared to the sham control group. Significant decreases in serum CK, LDH and AST activities were observed in the group of IPO rats, as compared to IR group.

Table 5 shows significantly decreased myocardium Na^+^-K^+^-ATPase and Ca^2+^-Mg^2+^-ATPase activities in the IR group as compared to the sham control group. Significant increases in myocardium Na^+^-K^+^-ATPase and Ca^2+^-Mg^2+^-ATPase activities were observed in the group of IPO rats, as compared to the IR group.

**Table 4:** Effect of ischemic postconditioning on serum CK, LDH and AST activities in IR rats.

Indexes	SO	IR	IPO
CK (U/L)	3,186.3 ± 351.6	7,319.2 ± 485.1 **	4,287.5 ± 332.8 ^##^
LDH (U/L)	1,528.4 ± 132.7	3,328.7 ± 295.3 **	1,832.8 ± 152.7 ^##^
AST (U/L)	251.8 ± 22.9	406.1 ± 31.8 **	298.6 ± 25.4 ^##^

** *p* < 0.01, IR group *vs*. SO group; ^##^ *p* < 0.01, IPO group *vs*. IR group.

**Table 5:** Effect of ischemic postconditioning on myocardium Na^+^-K^+^-ATPase and Ca^2+^-Mg^2+^-ATPase activities in IR rats.

Indexes	SO	IR	IPO
Na^+^-K^+^-ATPase (μmol pi/mg prot/h)	7.91 ± 0.77	4.82 ± 0.51 **	7.52 ± 0.67 ^##^
Ca^2+^-Mg^2+^-ATPase (μmol pi/mg prot/h)	8.52 ± 0.72	5.99 ± 0.46 **	7.61 ± 0.81 ^##^

** *p* < 0.01, IR group *vs*. SO group; ^##^ *p* < 0.01, IPO group *vs*. IR group.

Table 6 shows the influence of ischemic postconditioning on the levels of TBARS and the activities of myocardium SOD, CAT, GSH-Px and GR in control and experimental animals. IR rats showed significantly higher MDA levels as compared to sham control rats. IPO rats reduced the levels of lipid peroxidation markers. The activities of myocardium SOD, CAT, GSH-Px and GR were significantly lower in IR rats than in sham operation rats. Ischemic postconditioning significantly increased the activities of myocardium SOD, CAT, GSH-Px and GR in experimental animals.

**Table 6:** Effect of ischemic postconditioning on the levels of TBARS and the activities of myocardium SOD, CAT, GSH-Px and GR in IR rats.

Indexes	SO	IR	IPO
MDA	3.28 ± 0.26	6.82 ± 0.53 **	4.13 ± 0.29 ^##^
SOD	287.32 ± 31.73	98.41 ± 7.08 **	241.63 ± 27.56 ^##^
CAT	28.94 ± 2.54	12.62 ± 1.42 **	25.17 ± 2.64 ^##^
GSH-Px	37.82 ± 1.89	21.65 ± 2.65 **	34.06 ± 2.16 ^##^
GR	25.28 ± 1.66	10.63 ± 1.43 **	22.69 ± 1.98 ^##^

** *p* < 0.01, IR group *vs*. SO group; ^##^ *p* < 0.01, IPO group *vs*. IR group.

## 3. Discussion

In the present study, changes in the haemodynamic parameters supported the evidence of ischemic-reperfusion injury of the untreated rats’ hearts. Ventricular tachycardia (VT) was defined as sustained ventricular rate (absence of P wave, rapid, wide QRS complexes) greater than 1,000 beats/min with a reduction in arterial pressure below 40 mmHg. Ventricular fibrillation was defined as a ventricular rhythm without a recognizable QRS complex, in which signal morphology changed from cycle to cycle, and for which it was impossible to estimate heart rate. The value of VTt and VAS were significantly higher in IR rats than sham operation rats. This indicated that IR operation could increase ventricular tachycardia and fibrillation, exacerbate arrhythmia. In the IPO group, the value of VTt and VAS were significantly decreased. This indicated that IR operation could decrease ventricular tachycardia and fibrillation and alleviate arrhythmia. During ischemia, there was a significant fall in LVSP and LVEDP (an index for diastolic function) along with a tendency to fall of both left ventricular peak (+) dP/dt (an index for systolic function) and (−) dP/dt (an index for diastolic function). Following reperfusion, a further fall in LVSP and ±dp/dtmax was observed, along with an incomplete recovery of LVEDP. Reperfusion of the ischemic myocardium also caused a further significant reduction in both (+) and (−) dP/dt, which failed to recover during reperfusion. These indicated that IR decreased myocardium systolic and diastolic function. Similar findings have also been reported earlier [28,29,36,37]. The value of LVSP was significantly higher in IPO rats than IR rats. The value of LVEDP was significantly lower in IPO rats than IR rats. The value of ±dp/dtmax was significantly higher in IPO rats than IR rats. These results indicated that ischemic postconditioning significantly improved myocardium systolic and diastolic function in IR rats.

Reperfusion of the previously ischemic myocardium can cause tissue injury [24,27,33,36,38,39]. Galagudza *et al*. [37,40] suggested that the IPO could reduce infarct size in IR animals. In the current investigation, we adopted the strategy of post-conditioning, and found that ischemic postconditioning could reduce myocardial infarction area (49.42 ± 3.05 in IPO group (MIA) *vs.* 78.13 ± 4.17 (MIA) in IR group; 21.84 ± 1.56 in IPO group (MIA/AAR) *vs*. 39.07 ± 1.23 (MIA/AAR) in IR group). This indicated that ischemic postconditioning could provide protective effect against IR-induced myocardial injury. This conclusion confers the former investigators’ observations [33,36,38,39]. Intracellular markers, routinely determined by laboratory testing, are certain enzymes present at high activity in the tissue. The enzymes routinely measured in the clinical laboratory for the purpose of diagnosing and monitoring myocardial infarction include creatine kinase (CK), aspartate amino transferase (sGOT or AST), and lactate dehydrogenase (LDH) [38,39,41,42]. CK, AST and/or LDH isoenzyme determinations are useful when there is question about the tissue source of elevated enzyme activity. These enzymes are present in sufficiently high content in myocardial tissue so that the death of a relatively small amount of tissue results in a substantial increase in measurable enzyme activity in serum. The relatively high content of CK, AST and LDH in cardiac tissue suggests that the measure of these enzymes activity in serum is very useful to diagnosis and monitor myocardial infarction [40,41,43,44]. In the present study, LDH, AST, CK levels in serum of IPO rats were lower than those in the IR group. Ischemic postconditioning significantly reduced the release of intracellular enzymes (CK, AST, and LDH) from ischemic hearts, indicating a protection against cell membrane damage.

Na, K-ATPase (Na, K-pump) is an integral membrane protein that maintains normal physiological gradient of Na^+^ and K^+^ by catalyzing and transporting of these ions across the plasma membrane. The enzyme is comprised of a and b subunits, both of which are essential for ATPase and ion pumping function [42,43,44,45,46,47]. Additionally Na, K-ATPase acts as a signal transducer by interacting with neighboring membrane proteins. The binding of ouabain to the Na, K-pump evokes protein and lipid kinase cascades generating secondary messengers [43,44,46,47]. The Ca^2+^-ATPase is the major active calcium transport protein responsible for the maintenance of normal intracellular calcium levels in a variety of cell types. Maintenance of the cation gradient by Ca^2+^-ATPase is of fundamental importance in the control of hydration, volume, nutrient uptake and fluidity of cells, and is also essential for the contractility and excitability properties of muscles [45,46,48,49]. In the present study, myocardium Na^+^-K^+^-ATPase and Ca^2+^-Mg^2+^-ATPase activities was decreased in IR rats. Our findings are in agreement with previous observations showing that the membrane abnormalities in Na^+^-K^+^-ATPase, Na^+^-Ca^2+^ exchange and Ca^2+^-pump activities led to the occurrence of intracellular calcium overload in experimental rat models of myocardium ischemia reperfusion [47,50]. Significant increases in myocardium Na^+^-K^+^-ATPase and Ca^2+^-Mg^2+^-ATPase activities activities were observed in the group of IPO rats, as compared to IR group. We supposed that IPO may decrease Na^+^ to transfer into the cells, and promote its extracellular Mobile. As a result, this reduced intracellular Ca^2+^ content.

Early experimental findings have demonstrated the increased formation of oxygen-derived free radicals (oxy-radicals) in the myocardium during post-ischemic reperfusion [48,49,51,52]. Consistent with these, our findings showed that myocardial IR injury was accompanied by decreases in myocardial ATP generation capacity and antioxidant levels/activities, which are indirect indices of mitochondrial function and antioxidant status. While the decline in mitochondrial ATP generation capacity may be caused by oxidative damage on protein complexes involved in the electron transport process [50,51,52,53,54,55,56], the inhibition of antioxidant enzymes, such as SOD, CAT, GSH-Px and GR can aggravate the myocardial oxidative injury. ROS produced with reperfusion are scavenged by the antioxidant enzymes SOD, GSH-Px, CAT and GR. We studied SOD, GSH-Px, CAT and GR to investigate the enzymatic antioxidant status of rat heart after IR. Significantly decreased activities of SOD, GSH-Px, CAT and GR were observed in the IR group in comparison with sham group. Ischemic postconditioning significantly increased the activities of myocardium SOD, CAT, GSH-Px and GR in experimental animals. This indicates that ischemic postconditioning may alleviate oxidative injury in IR rats.

## 4. Experimental

### 4.1. Cardiac Ischemic Reperfusion Models

Wistar rats were provided by the Fengxian Hospital Animal Lab Centre (Shanghai, China). Forty-eight rats of either sex, with body weight of 250 g ± 20 g, were randomly assigned to three groups (16 in each): sham operation group (SO group), ischemia/reperfusion group (IR group), and ischemic postconditioning group (IPO group). Before the experiment, the rats were abstained from food for 12 h but free to drink water at any time. Briefly, after nembutal anaesthesia (40 mg/kg ip), the animals were intubated and mechanical ventilation was achieved with a positive rodent respirator using atmospheric air at a tidal volume of 5 mL and a rate of about 50 breaths/minute. The heart was exposed through a left thoracotomy in the third intercostal space. A 6.0 silk non-traumatic suture was passed through the epicardial layer around the major branch of the left coronary artery, about 2 mm of its origin. A small plastic button (diameter about 5 mm) was threaded through the ligature and placed in contact with the heart. The ends were passed through small vinyl tube and exteriorized. The heart was replaced in the chest and the chest closed after removing the residual air to avoid pneumothorax. The occlusion of the artery was produced by applying tension to the ligature, and reperfusion was achieved by releasing the tension. The method is very convenient for producing ischemia/reperfusion injury in late preconditioning (24 h after the surgery and the acute preconditioning). The electrocardiogram (ECG) was monitored throughout the experiment. All intravenous applications of saline or drugs were performed through the tail vein. The surgical mortality rate of the above procedure was very low (1–3%).

Rats in SO group were allowed to a continuous perfusion for 150 min. IR group rats were kept ischemic for 30 min and reperfused for 120 min. IPO group rats were maintained ischemia for 30 min, then postconditioning was performed by three cycles of 30-second reperfusion and 30-second ischemia, and finally reperfused for 120 min. 

### 4.2. Myocardial Infarct Size Determination

At the end of the 24-h reperfusion period, rats were reanesthetized, the coronary artery was reoccluded at the previous site of occlusion, and the heart was excised after Evans blue perfusion. The area at risk was identified by Evans blue staining, and the infarct area was identified by 2,3,5-triphenyltetrazolium chloride (TTC) staining. The area at risk was identified as the nonblue region and expressed as a percentage of the left ventricle area. The infarcted area was identified as the TTC-negative zone and expressed as a percentage of the area at risk.

### 4.3. Tissue Preparation

Fresh ventricular tissues were placed in medium containing (in mM): 220 mannitol, 70 sucrose, 10 HEPES, 1 EGTA, 1 PMSF, 1 sodium orthovanadate, 5 sodium fluoride, and 1 Na_2_ β-glycerol phosphate (Sigma-Aldrich, St. Louis, MO, USA), with 5 μL/mL of protease inhibitor cocktail, pH 7.4 at 4 °C. The tissues were scissor minced and homogenized on ice with a Teflon Potter homogenizer. The homogenate was centrifuged at 17,600 g for 30 min at 4 °C to collect the cytosol.

### 4.4. Biochemical Analysis

CK, LDH, AST in blood were measured using commercially available kits.

Na^+^-K^+^-ATPase and Ca^2+^-Mg^2+^-ATPase activities were assayed by spectrophotometrically measuring the amount of inorganic phosphate liberated following incubation of the tissue extract with disodium ATP (Sigma, Welwyn Garden City, UK) as in previous studies [54,57]. 

The lipid peroxidation level in heart homogenate was measured as MDA which is the end product of lipid peroxidation, and reacts with thiobarbituric acid (TBA) as a TBA reactive substance (TBARS) to produce a red coloured complex which has peak absorbance at 532 nm according to Buege and Aust [55,58]. A total of 125 mL of supernatants were homogenized by sonication with TBS (50 mL), TCA-BHT (butylhydroxytoluene, 125 mL) in order to precipitate proteins and then centrifuged (1,000 g, 10 min, 4 °C). In total, 200 mL of supernatant were mixed with HCl (40 mL, 0.6 M) and TBA dissolved in Tris (160 mL, 0.7 M) and the mixture was heated at 80 °C for 10 min. The absorbance of the resultant supernatant was read at 530 nm. The amount of TBARS was calculated by using an extinction coefficient of 156 mM^−1^ ^cm−1^.

SOD activity was measured according to the method of McCord and Fridowich [56,59], based on the production of superoxide radicals during the conversion of xanthine to uric acid by xanthine oxidase and the inhibition of cytochrome C reduction. One unit of SOD acitivity was defined as the amount of SOD that produces 50% inhibition of cytochrome C reduction. CAT and GPx activity were determined following the methods of Clairbone [57,60], and of Gunzler *et al*. [58,61], respectively. Glutathione reductase (GR) activity was assayed as described by Calberg and Mannervik [59,62], with some modifications, by measuring the oxidation of NADPH at 340 nm. The reaction mixture consisted of 0.1 M sodium phosphate buffer (pH 7.5), 1 mM EDTA, 0.63 mM NADPH and 0.15 mM GSSG.

## 5. Conclusions

The present study demonstrates that ischemic postconditioning can improve arrhythmia cordis, decrease myocardial necrosis and oxidative injury in IR rats. There are the limitations in the translation of rat data to humans, with respect to confounders such as species [60,63], age [61,64] and co-morbidities/co-medications [3,62,65,66,67].
